# Supplementary material for: The Relation between Vitamin D Level and Lung Clearance Index in Cystic Fibrosis—A Pilot Study
Source: Children (Basel). 2022 Mar 1;9(3):329. doi: 10.3390/children9030329 (PMC8947157; doi:10.3390/children9030329)
Supplement: Supplementary file 1 [file children-09-00329-s001.zip › children-1605466-supplementary.pdf]

Supplementary Materials

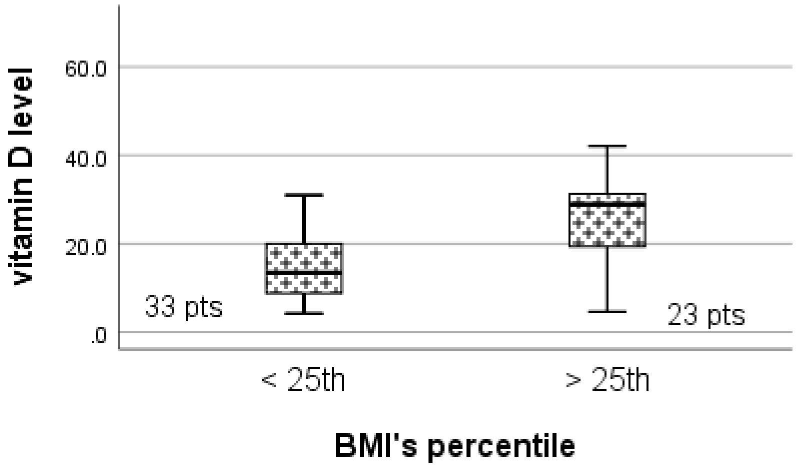

Figure S1. Vitamin D level according to BMI percentile.

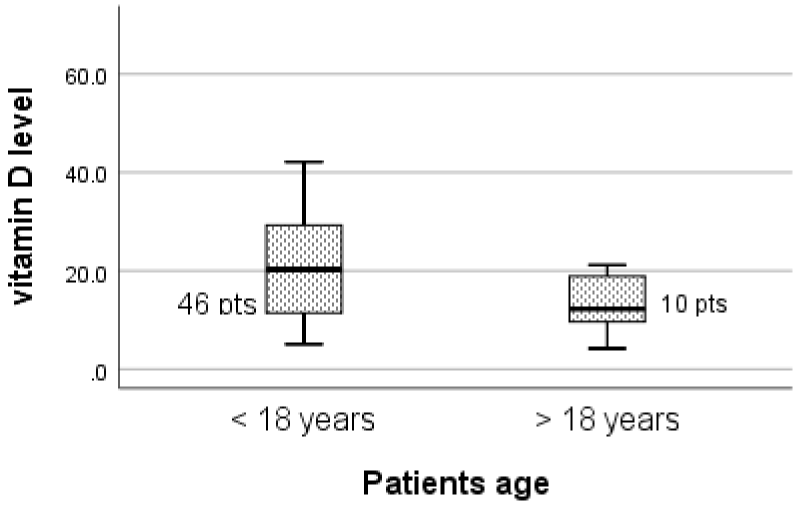

Figure S2. Vitamin D level according to patients age.

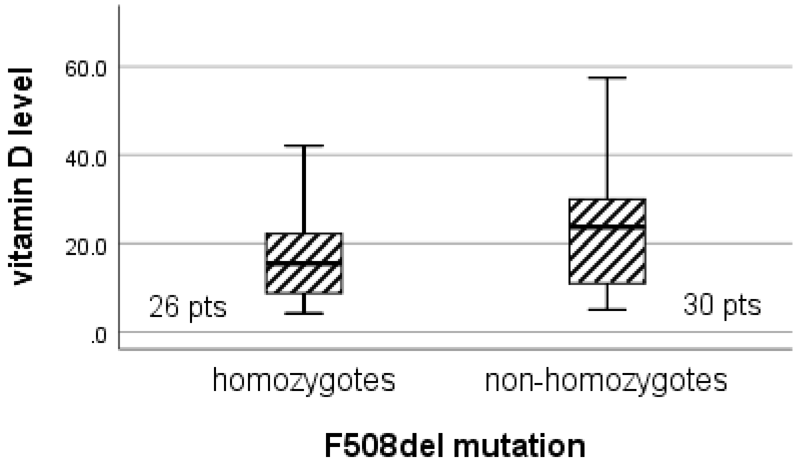

Figure S3. Vitamin D level according to F508del mutation.

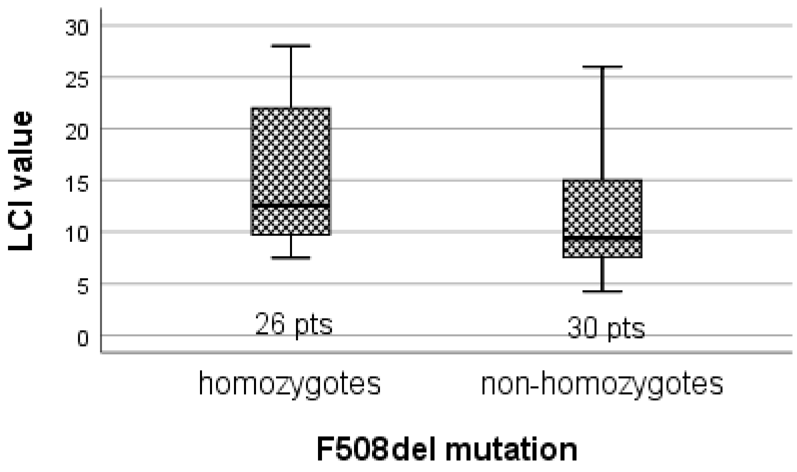

Figure S4. LCI value according to F508del mutation.

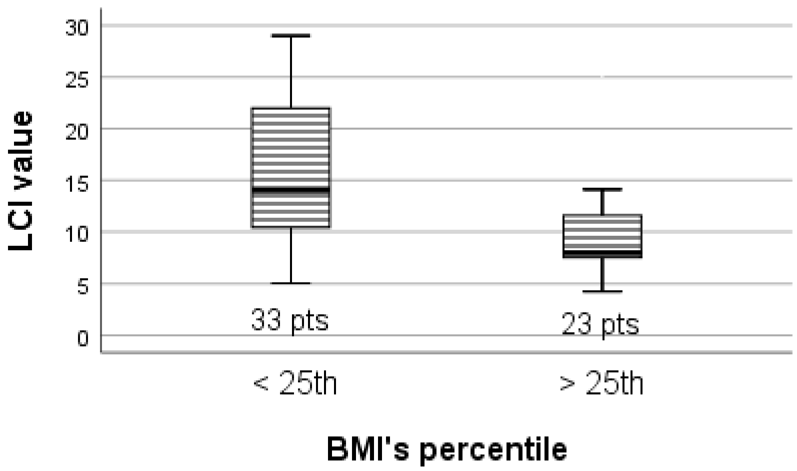

Figure S5. LCI value according to BMI percentile.

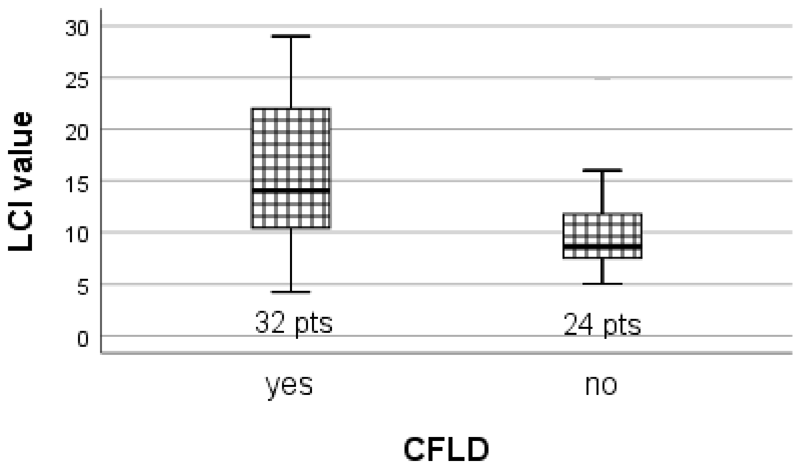

Figure S6. LCI value according to CFLD.

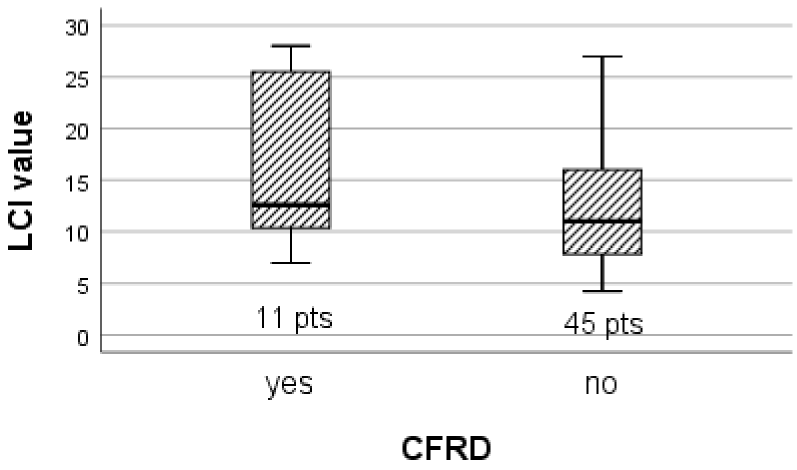

Figure S7. LCI value according to CFRD.

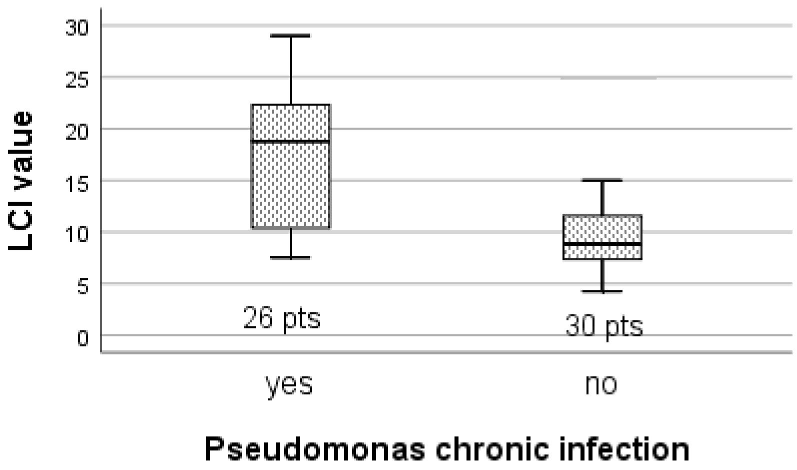

Figure S8. LCI value according to Pse chronic infection.
